# 4-(4-Hydroxy­phenyl­diazen­yl)­benzonitrile

**DOI:** 10.1107/S1600536808004716

**Published:** 2008-02-22

**Authors:** Chao Zhi Zhang

**Affiliations:** a Ordered Matter Science Research Center, College of Chemistry and Chemical Engineering, Southeast University, Nanjing 210096, People’s Republic of China

## Abstract

The molecule of the title compound, C_13_H_9_N_3_O, is achiral but forms a chiral arrangement in the crystal structure. The mol­ecule adopts an *E* configuration with respect to the N=N bond and is almost planar, with an r.m.s. deviation of 0.0439 Å from the plane through all atoms in the mol­ecule. The dihedral angle between the two benzene rings is 2.2 (2)°. In the crystal structure, inter­molecular O—H⋯N hydrogen bonding generates a chain.

## Related literature

For the preparation of tetra­zole derivatives from nitrile compounds, see: Dunica *et al.* (1991 ▶); Wittenberger & Donner (1993 ▶). For the general chemistry of tetra­zole compounds, see: Xiong *et al.* (2002 ▶). For a similar structure, see: Harada *et al.* (1997 ▶).
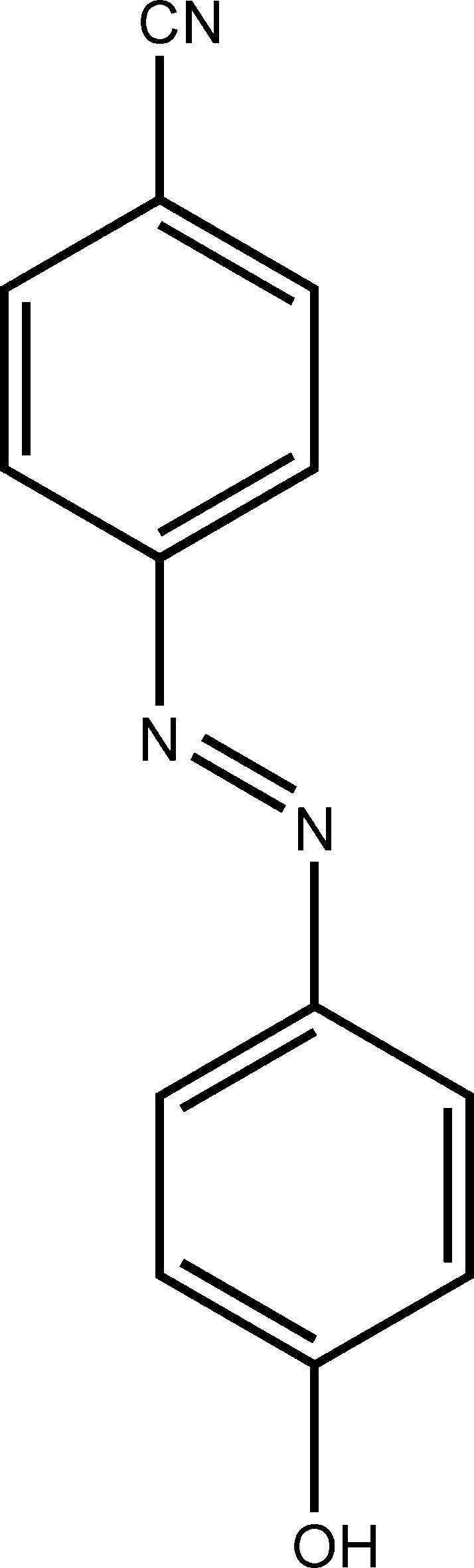


## Experimental

### 

#### Crystal data

C_13_H_9_N_3_O
*M* *_r_* = 223.23
Monoclinic, 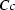

*a* = 6.5307 (13) Å
*b* = 10.747 (2) Å
*c* = 15.851 (3) Å
β = 93.54 (3)°
*V* = 1110.4 (4) Å^3^
*Z* = 4
Mo *K*α radiation
μ = 0.09 mm^−1^
*T* = 293 (2) K
0.18 × 0.05 × 0.05 mm

#### Data collection

Rigaku Mercury2 diffractometer
Absorption correction: multi-scan (*CrystalClear*; Rigaku, 2005 ▶) *T*_min_ = 0.781, *T*_max_ = 1 (expected range = 0.778–0.996)
5599 measured reflections
2532 independent reflections
1296 reflections with *I* > 2σ(*I*)
*R*_int_ = 0.073

#### Refinement

*R*[*F* ^2^ > 2σ(*F* ^2^)] = 0.053
*wR*(*F* ^2^) = 0.145
*S* = 0.97
1278 reflections
154 parameters
2 restraints
H-atom parameters constrained
Δρ_max_ = 0.17 e Å^−3^
Δρ_min_ = −0.19 e Å^−3^

### 

Data collection: *CrystalClear* (Rigaku, 2005 ▶); cell refinement: *CrystalClear*; data reduction: *CrystalClear*; program(s) used to solve structure: *SHELXS97* (Sheldrick, 2008 ▶); program(s) used to refine structure: *SHELXL97* (Sheldrick, 2008 ▶); molecular graphics: *SHELXTL* (Sheldrick, 2008 ▶); software used to prepare material for publication: *SHELXTL*.

## Supplementary Material

Crystal structure: contains datablocks I, global. DOI: 10.1107/S1600536808004716/kp2153sup1.cif

Structure factors: contains datablocks I. DOI: 10.1107/S1600536808004716/kp2153Isup2.hkl

Additional supplementary materials:  crystallographic information; 3D view; checkCIF report

## Figures and Tables

**Table 1:** Hydrogen-bond geometry (Å, °)

*D*—H⋯*A*	*D*—H	H⋯*A*	*D*⋯*A*	*D*—H⋯*A*
O1—H1*A*⋯N1^i^	0.85	2.01	2.817 (5)	159

Symmetry code: (i) 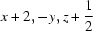.

**Fig. 1.**
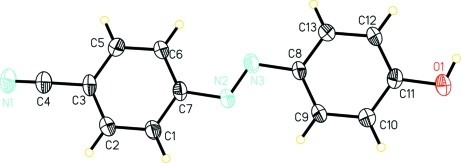
A view of the title compound with the atomic numbering scheme. Displacement ellipsoids are drawn at the 30% probability level.

**Fig. 2.**
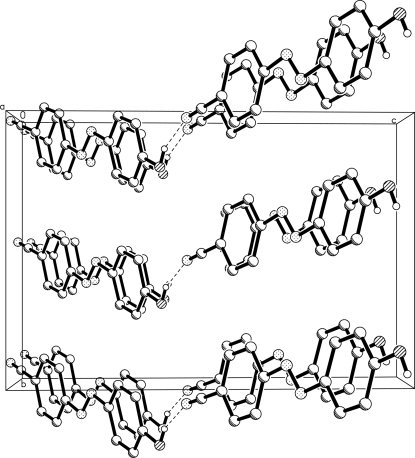
The crystal packing of the title compound viewed along the a axis. Hydrogen atoms not involved in hydrogen bonding (dashed lines) were omitted for clarity.

## supplementary crystallographic information

### Comment

Nitrile compounds are the precursor of tetrazole derivatives (Dunica, *et al.*, 1991; Wittenberger, *et al.*, 1993) and can be used for the design of noncentrosymmetric bulk materials (Xiong, *et al.*(2002)). We report here the crystal structure of the title compound, 4-(4-hydroxyphenylazo)benzonitrile, (I) (Fig. 1).

In I, the N=N double bond [1.257 (4) Å] is in the range found in other similar azo complexes with a *trans*-configuration (Harada, *et al.*, 1997). The torsion angle C7 - N2 - N3 - C8 is -179.30 (0.32)°. The dihedral angle between the two benzene rings is 2.18 (1/5) °. The crystal structure involves O—H···N hydrogen bond resulting in the formation of a chain.

### Experimental

A solution of 4-cyanoaniline (0.71 g, 6 mmol) in a solution of hydrochloric acid (6 ml, 4*M*) was added to a solution of sodium nitrite (0.42 g, 6.1 mmol) in 2 ml water, and the mixture was stirred for 4 h under N2 atmosphere at 273–278 K. Then urea (0.01 g, 0.2 mmol) was added to decompose excessive nitrous acid, and the mixture was further stirred for 30 min. The solution of the diazonium salt was added to a aqueous phenol (0.62 g, 6.6 mmol), sodium carbonate (3 g), baking soda (0.2 g) and ice (15 g) at 273–278 K. The mixture was stirred for 7 h. After the reaction solution was neutralized with a solution of hydrochoric acid (13.6 ml, 3 *M*), the mixture was filtrated. A yellow block-like crystals (1.27 g, 5.7 mmol, 95%), which is suitable for X-ray analysis, were obtained by recrystallization from ethyl acetate (18 ml).

### Refinement

Positional parameters of all the H atoms were calculated geometrically and were allowed to ride on the C atoms to which they are bonded, with *U*_iso_(H) = 1.2Ueq(C),orUiso(H) = 1.2Ueq(O). In the absence of significant anomalous scattering effects the Friedel pairs were merged.

### Crystal data

**Table:**

C_13_H_9_N_3_O	*F*_000_ = 464
*M**_r_* = 223.23	*D*_x_ = 1.335 Mg m^−^^3^
Monoclinic, *C**c*	Mo *K*α radiation λ = 0.71073 Å
Hall symbol: C -2yc	Cell parameters from 4352 reflections
*a* = 6.5307 (13) Å	θ = 3.7–27.7º
*b* = 10.747 (2) Å	µ = 0.09 mm^−^^1^
*c* = 15.851 (3) Å	*T* = 293 (2) K
β = 93.54 (3)º	BLOCK, yellow
*V* = 1110.4 (4) Å^3^	0.18 × 0.05 × 0.05 mm
*Z* = 4	

### Data collection

**Table:**

Rigaku Mercury2 diffractometer	2532 independent reflections
Radiation source: fine-focus sealed tube	1296 reflections with *I* > 2σ(*I*)
Monochromator: graphite	*R*_int_ = 0.073
Detector resolution: 13.6612 pixels mm^-1^	θ_max_ = 27.5º
*T* = 293(2) K	θ_min_ = 3.7º
ω scans	*h* = −8→8
Absorption correction: multi-scan(CrystalClear; Rigaku, 2005)	*k* = −13→13
*T*_min_ = 0.781, *T*_max_ = 1	*l* = −20→20
5599 measured reflections	

### Refinement

**Table:**

Refinement on *F*^2^	Secondary atom site location: difference Fourier map
Least-squares matrix: full	Hydrogen site location: inferred from neighbouring sites
*R*[*F*^2^ > 2σ(*F*^2^)] = 0.053	H-atom parameters constrained
*wR*(*F*^2^) = 0.145	*w* = 1/[σ^2^(*F*_o_^2^) + (0.0762*P*)^2^] where *P* = (*F*_o_^2^ + 2*F*_c_^2^)/3
*S* = 0.98	(Δ/σ)_max_ < 0.001
1278 reflections	Δρ_max_ = 0.17 e Å^−^^3^
154 parameters	Δρ_min_ = −0.19 e Å^−^^3^
2 restraints	Extinction correction: none
Primary atom site location: structure-invariant direct methods	Absolute structure: indeterminate

### Special details

**Table:**

Geometry. All e.s.d.'s (except the e.s.d. in the dihedral angle between two l.s. planes) are estimated using the full covariance matrix. The cell e.s.d.'s are taken into account individually in the estimation of e.s.d.'s in distances, angles and torsion angles; correlations between e.s.d.'s in cell parameters are only used when they are defined by crystal symmetry. An approximate (isotropic) treatment of cell e.s.d.'s is used for estimating e.s.d.'s involving l.s. planes.
Refinement. Refinement of *F*^2^ against ALL reflections. The weighted *R*-factor *wR* and goodness of fit *S* are based on *F*^2^, conventional *R*-factors *R* are based on *F*, with *F* set to zero for negative *F*^2^. The threshold expression of *F*^2^ > σ(*F*^2^) is used only for calculating *R*-factors(gt) *etc*. and is not relevant to the choice of reflections for refinement. *R*-factors based on *F*^2^ are statistically about twice as large as those based on *F*, and *R*- factors based on ALL data will be even larger.

### Fractional atomic coordinates and isotropic or equivalent isotropic displacement parameters (Å^2^)

**Table:**

	*x*	*y*	*z*	*U*_iso_*/*U*_eq_	
N3	0.2824 (5)	0.0455 (4)	0.1896 (2)	0.0497 (10)	
N2	0.1650 (5)	0.1302 (4)	0.1608 (2)	0.0526 (11)	
C8	0.4581 (6)	0.0892 (5)	0.2376 (3)	0.0447 (11)	
O1	0.9855 (5)	0.1997 (4)	0.3729 (2)	0.0717 (11)	
H1A	1.0618	0.1372	0.3848	0.108*	
C3	−0.3689 (6)	0.0230 (5)	0.0211 (3)	0.0493 (12)	
C7	−0.0130 (6)	0.0862 (4)	0.1136 (3)	0.0457 (11)	
C12	0.7767 (7)	0.0358 (5)	0.3110 (3)	0.0502 (11)	
H12A	0.8709	−0.0242	0.3303	0.060*	
C4	−0.5570 (7)	−0.0106 (5)	−0.0251 (3)	0.0572 (13)	
C6	−0.0538 (7)	−0.0375 (5)	0.0957 (3)	0.0543 (13)	
H6A	0.0385	−0.0988	0.1146	0.065*	
C2	−0.3273 (7)	0.1461 (5)	0.0391 (3)	0.0583 (14)	
H2B	−0.4188	0.2075	0.0197	0.070*	
C5	−0.2315 (6)	−0.0695 (5)	0.0497 (3)	0.0565 (14)	
H5A	−0.2600	−0.1526	0.0377	0.068*	
C9	0.4922 (6)	0.2126 (4)	0.2580 (3)	0.0577 (13)	
H9A	0.3959	0.2725	0.2407	0.069*	
C11	0.8129 (7)	0.1589 (4)	0.3289 (3)	0.0513 (12)	
C13	0.6011 (7)	0.0010 (5)	0.2644 (3)	0.0519 (13)	
H13A	0.5788	−0.0823	0.2511	0.062*	
C10	0.6682 (7)	0.2471 (5)	0.3040 (3)	0.0622 (15)	
H10A	0.6898	0.3302	0.3183	0.075*	
N1	−0.7085 (7)	−0.0361 (5)	−0.0604 (3)	0.0712 (12)	
C1	−0.1502 (7)	0.1783 (5)	0.0860 (3)	0.0580 (14)	
H1B	−0.1228	0.2613	0.0990	0.070*	

### Atomic displacement parameters (Å^2^)

**Table:**

	*U*^11^	*U*^22^	*U*^33^	*U*^12^	*U*^13^	*U*^23^
N3	0.041 (2)	0.055 (3)	0.051 (2)	0.000 (2)	−0.0098 (18)	0.0033 (19)
N2	0.043 (2)	0.054 (3)	0.059 (3)	0.002 (2)	−0.0175 (19)	0.001 (2)
C8	0.031 (2)	0.051 (3)	0.051 (3)	0.0005 (19)	−0.008 (2)	0.000 (2)
O1	0.055 (2)	0.071 (3)	0.084 (3)	0.0087 (18)	−0.0356 (19)	−0.014 (2)
C3	0.035 (2)	0.070 (4)	0.041 (2)	−0.005 (2)	−0.0080 (19)	0.000 (2)
C7	0.042 (3)	0.055 (3)	0.039 (3)	−0.002 (2)	−0.010 (2)	0.003 (2)
C12	0.039 (3)	0.055 (3)	0.055 (3)	0.008 (2)	−0.012 (2)	0.003 (2)
C4	0.046 (3)	0.072 (4)	0.052 (3)	−0.006 (3)	−0.006 (2)	−0.001 (2)
C6	0.045 (3)	0.053 (3)	0.063 (3)	0.007 (2)	−0.017 (2)	−0.004 (3)
C2	0.054 (3)	0.057 (3)	0.061 (3)	0.006 (2)	−0.019 (2)	0.003 (2)
C5	0.053 (3)	0.054 (3)	0.060 (3)	0.000 (2)	−0.009 (2)	−0.012 (2)
C9	0.045 (3)	0.049 (3)	0.076 (4)	0.007 (2)	−0.023 (2)	0.001 (3)
C11	0.046 (3)	0.057 (3)	0.049 (3)	−0.003 (2)	−0.010 (2)	−0.006 (2)
C13	0.045 (3)	0.049 (3)	0.061 (3)	0.008 (2)	−0.007 (2)	−0.007 (2)
C10	0.055 (3)	0.050 (3)	0.078 (4)	0.005 (2)	−0.021 (3)	−0.004 (3)
N1	0.048 (2)	0.091 (3)	0.072 (3)	−0.013 (2)	−0.017 (2)	−0.007 (2)
C1	0.051 (3)	0.049 (3)	0.071 (4)	−0.004 (2)	−0.021 (3)	0.008 (2)

### Geometric parameters (Å, °)

**Table:**

N3—N2	1.258 (4)	C12—H12A	0.9300
N3—C8	1.417 (5)	C4—N1	1.140 (6)
N2—C7	1.424 (5)	C6—C5	1.375 (7)
C8—C13	1.380 (6)	C6—H6A	0.9300
C8—C9	1.380 (6)	C2—C1	1.380 (6)
O1—C11	1.361 (5)	C2—H2B	0.9300
O1—H1A	0.8501	C5—H5A	0.9300
C3—C2	1.377 (7)	C9—C10	1.373 (6)
C3—C5	1.395 (7)	C9—H9A	0.9300
C3—C4	1.437 (6)	C11—C10	1.379 (7)
C7—C6	1.382 (6)	C13—H13A	0.9300
C7—C1	1.388 (6)	C10—H10A	0.9300
C12—C11	1.371 (7)	C1—H1B	0.9300
C12—C13	1.377 (7)		
			
N2—N3—C8	114.2 (3)	C3—C2—H2B	120.0
N3—N2—C7	114.2 (3)	C1—C2—H2B	120.0
C13—C8—C9	119.4 (4)	C6—C5—C3	119.9 (4)
C13—C8—N3	116.6 (4)	C6—C5—H5A	120.0
C9—C8—N3	124.0 (4)	C3—C5—H5A	120.0
C11—O1—H1A	108.4	C10—C9—C8	120.1 (4)
C2—C3—C5	120.1 (4)	C10—C9—H9A	119.9
C2—C3—C4	119.9 (4)	C8—C9—H9A	119.9
C5—C3—C4	119.9 (5)	O1—C11—C12	122.9 (4)
C6—C7—C1	120.6 (4)	O1—C11—C10	117.2 (4)
C6—C7—N2	124.6 (4)	C12—C11—C10	119.9 (4)
C1—C7—N2	114.8 (4)	C12—C13—C8	120.3 (5)
C11—C12—C13	120.0 (4)	C12—C13—H13A	119.8
C11—C12—H12A	120.0	C8—C13—H13A	119.8
C13—C12—H12A	120.0	C9—C10—C11	120.2 (4)
N1—C4—C3	178.5 (6)	C9—C10—H10A	119.9
C5—C6—C7	119.7 (4)	C11—C10—H10A	119.9
C5—C6—H6A	120.2	C2—C1—C7	119.6 (5)
C7—C6—H6A	120.2	C2—C1—H1B	120.2
C3—C2—C1	120.0 (4)	C7—C1—H1B	120.2
			
C7—N2—N3—C8	−179.2 (4)		

### Hydrogen-bond geometry (Å, °)

**Table:**

*D*—H···*A*	*D*—H	H···*A*	*D*···*A*	*D*—H···*A*
O1—H1A···N1^i^	0.85	2.01	2.817 (5)	159

Symmetry codes: (i) *x*+2, −*y*, *z*+1/2.
